# Supplementary figures and images for: Inhibition of calcineurin by FK506 stimulates germinal vesicle breakdown of mouse oocytes in hypoxanthine-supplemented medium
Source: PeerJ. 2017 Feb 23;5:e3032. doi: 10.7717/peerj.3032 (PMC5326542; doi:10.7717/peerj.3032)

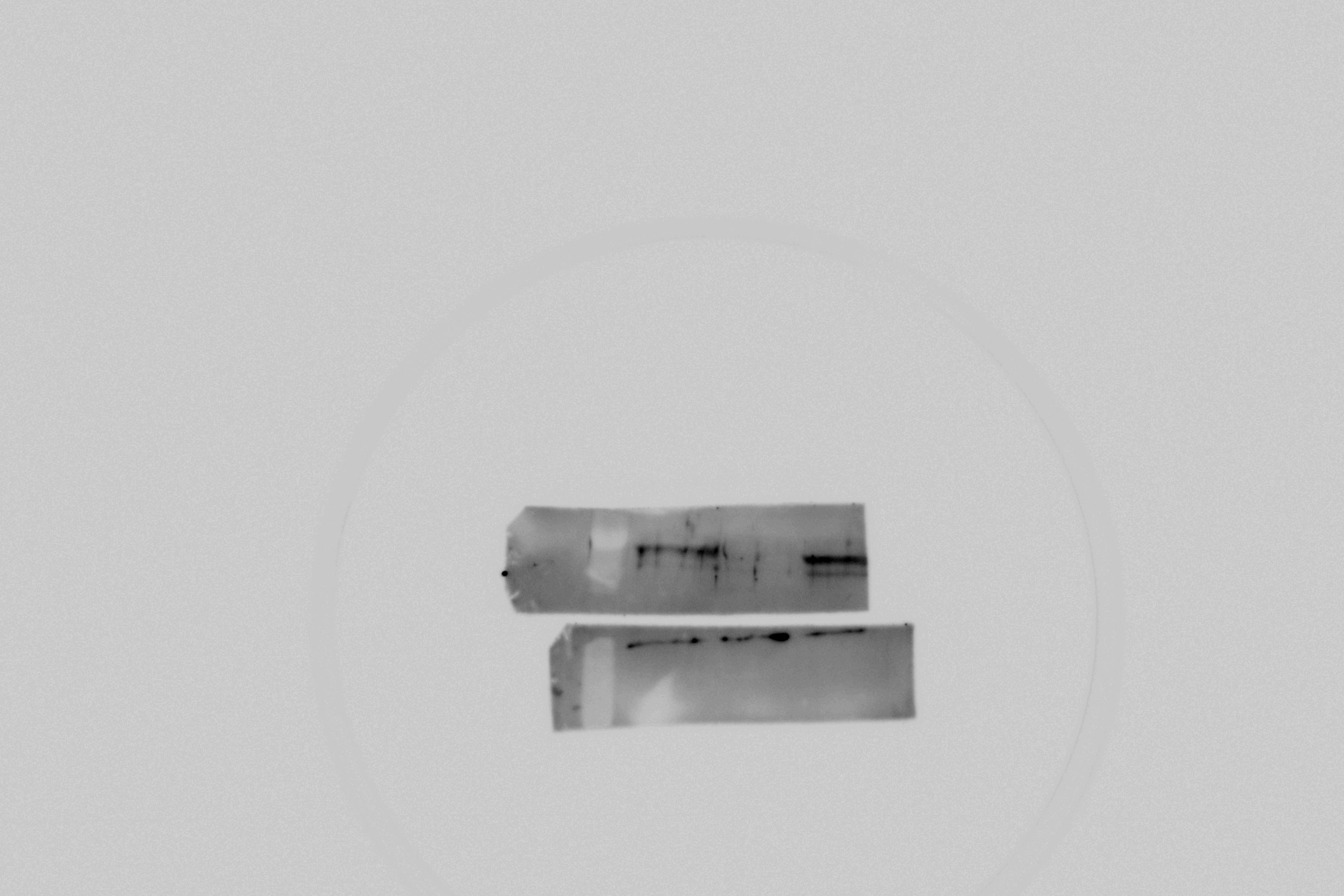

Supplement: Supplemental Information 1 — The original figure of Western Blotting in Fig. 2C. [file peerj-05-3032-s002.zip › Figure2 supplement/C.jpg]
